# Whole body MRI: Improved Lesion Detection and Characterization With Diffusion Weighted Techniques

**DOI:** 10.1002/jmri.24285

**Published:** 2013-08-19

**Authors:** Rajpaul Attariwala, Wayne Picker

**Affiliations:** AIM Medical ImagingVancouver, BC, Canada

**Keywords:** MRI; whole body MRI, diffusion-weighted imaging DWI, oncology imaging, b-value, apparent diffusion coefficient ADC

## Abstract

Diffusion-weighted imaging (DWI) is an established functional imaging technique that interrogates the delicate balance of water movement at the cellular level. Technological advances enable this technique to be applied to whole-body MRI. Theory, b-value selection, common artifacts and target to background for optimized viewing will be reviewed for applications in the neck, chest, abdomen, and pelvis. Whole-body imaging with DWI allows novel applications of MRI to aid in evaluation of conditions such as multiple myeloma, lymphoma, and skeletal metastases, while the quantitative nature of this technique permits evaluation of response to therapy. Persisting signal at high b-values from restricted hypercellular tissue and viscous fluid also permits applications of DWI beyond oncologic imaging. DWI, when used in conjunction with routine imaging, can assist in detecting hemorrhagic degradation products, infection/abscess, and inflammation in colitis, while aiding with discrimination of free fluid and empyema, while limiting the need for intravenous contrast. DWI in conjunction with routine anatomic images provides a platform to improve lesion detection and characterization with findings rivaling other combined anatomic and functional imaging techniques, with the added benefit of no ionizing radiation.

ONGOING TECHNOLOGICAL AND computer processing advances with MRI have ushered in a new era of whole-body imaging. Using multi-station, or even continuous moving table technology allows complete imaging from the head to foot, with conventional anatomic sequences such as T1, T2, STIR, and angiographic protocols routinely being performed 1–5. These morphologic techniques take advantage of MRI’s exquisite contrast and spatial resolution to provide great detail of the whole-body anatomy and organ specific tissue composition. A relatively new MRI sequence that takes advantage of these recent technological advances is diffusion-weighted imaging (DWI). This functional technique has long been used in the brain to demonstrate alteration in intra-and extracellular water content from disruption of the transmembrane water flux. These cellular level alterations are visible before changes can be identified on morphologic routine sequences. This technique in the brain was first described by LeBihan and Breton 6, and is routinely used in assessment for early ischemia. Application of DWI to the body is more complex secondary to the greater variability of tissue body water composition and due to the complexity imparted from organ movement. Technological advances such as parallel imaging, echo planar imaging, and multichannel receiver coils have enabled these difficulties to be overcome, allowing high-quality repeatable application of DWI to the whole body 7,8.

Whole-body MRI has continually evolved as a detection method for a variety of pathologic processes. In addition to vascular applications, it has been used for detection of unknown primary malignancy, determining skeletal spread of metastasis, and as a cancer screening and staging tool 1,3,9.

## BACKGROUND CONSIDERATIONS FOR CLINICAL WHOLE-BODY DIFFUSION

DWI with MRI was first described by Stejskal and Tanner 10 and is based on steady-state kinetic Brownian motion of a solute in a solvent. Einstein in 1905 11 applied this principle to a Stokes particle in quiescent fluid at a uniform temperature, which resulted in the Stokes-Einstein Eq. [1] upon which diffusion weighting imaging is based.
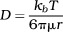
[1]

For body temperature and based on semisolid tissue, k_b_ and T are constants, while r represents a solute molecule subject to viscosity μ. When applied to the body, r, is on the same order of magnitude as a cell, and thus diffusion represents an effective mean free path of water in both the intracellular and extracellular environment. For the application of diffusion to the body, unlike the central nervous system, water is not as tightly regulated. In the body, water movement is both flow and tissue dependent, and the fat signal needs to be eliminated to have a rapid water selective sequence. Various fat suppression techniques have been used, with nonselective STIR sequences being commonly used at for whole body at 1.5 Tesla (T) 12,13. Areas of incomplete fat saturation commonly occur at edge of field and where the prescan radiofrequency (RF) map is inhomogeneous. These areas are commonly seen at the neck/shoulder interface, at fat/air gaps in the bowel/low pelvis, between the thighs and adjacent to pendulous breast tissue. These areas frequently have restricted signal changes and thus reviewing diffusion images in conjunction with conventional MRI sequences is invaluable. These errors can also be minimized by shortening the z-axis imaged field of view to optimize imaging around isocenter, and by optimizing the receiver bandwidth 13.

**Figure 1:**
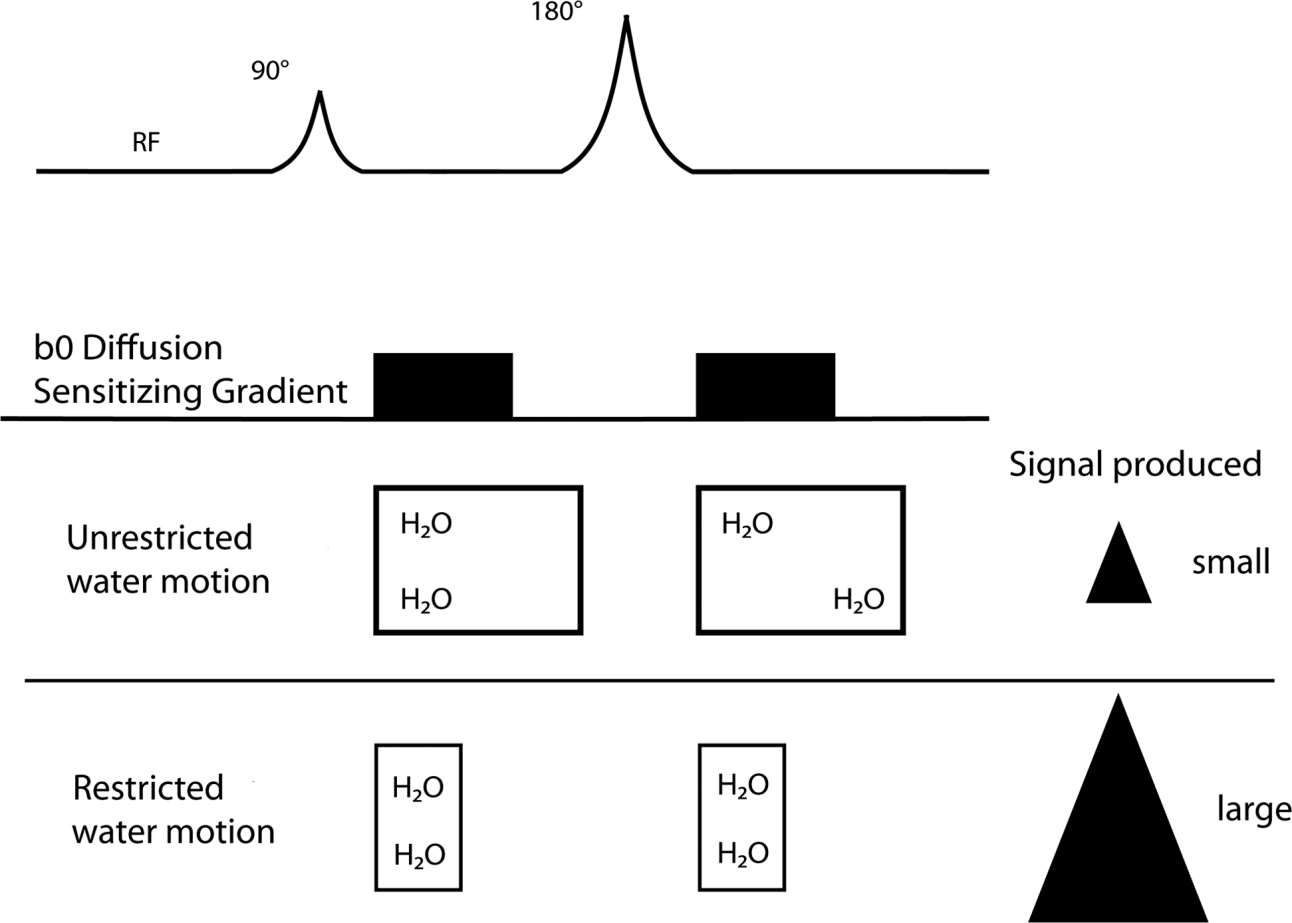
Application of water motion probing diffusion sensitizing gradients between polarizing RF pulses. Free unrestricted water movement in between application of the diffusion sensitizing gradients results in low signal. Increased signal is produced in tissue where water is restricted from moving between the two motion probing diffusion sensing gradient applications (bottom row).

**Figure 2:**
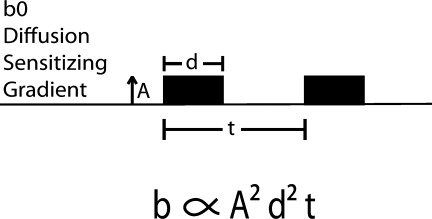
b-value, or diffusion gradient field strength. It is dependent on gradient field strength amplitude, A, duration of application of the diffusion gradient, d, and the time interval between applying the two motion probing pulses, t.

DWI with fat background suppression (DWIBS), was first applied to whole-body MRI imaging by Takahara et al 12, and is based on water motion probing gradients. Motion probing gradients are a method of nondirectionally sensitizing water to determine the water movement between diffusion sensitizing gradient pulses (Fig. 1). If water moves substantially between diffusion sensitizing gradients, the resulting bulk water signal would be low; however, if water is restricted from moving between diffusion sensitizing gradients, the signal would be high. The diffusion gradient strength, b-value [s/mm^2^], was first described by LeBihan in 1985 6. The b-value is dependent on the duration of the diffusion sensitizing gradient, amplitude, and time between application of the sensitizing gradient. Typically, to increase the b-value, greater amplitude of the diffusion sensitizing gradient is applied (Fig. 2).

**Figure 3:**
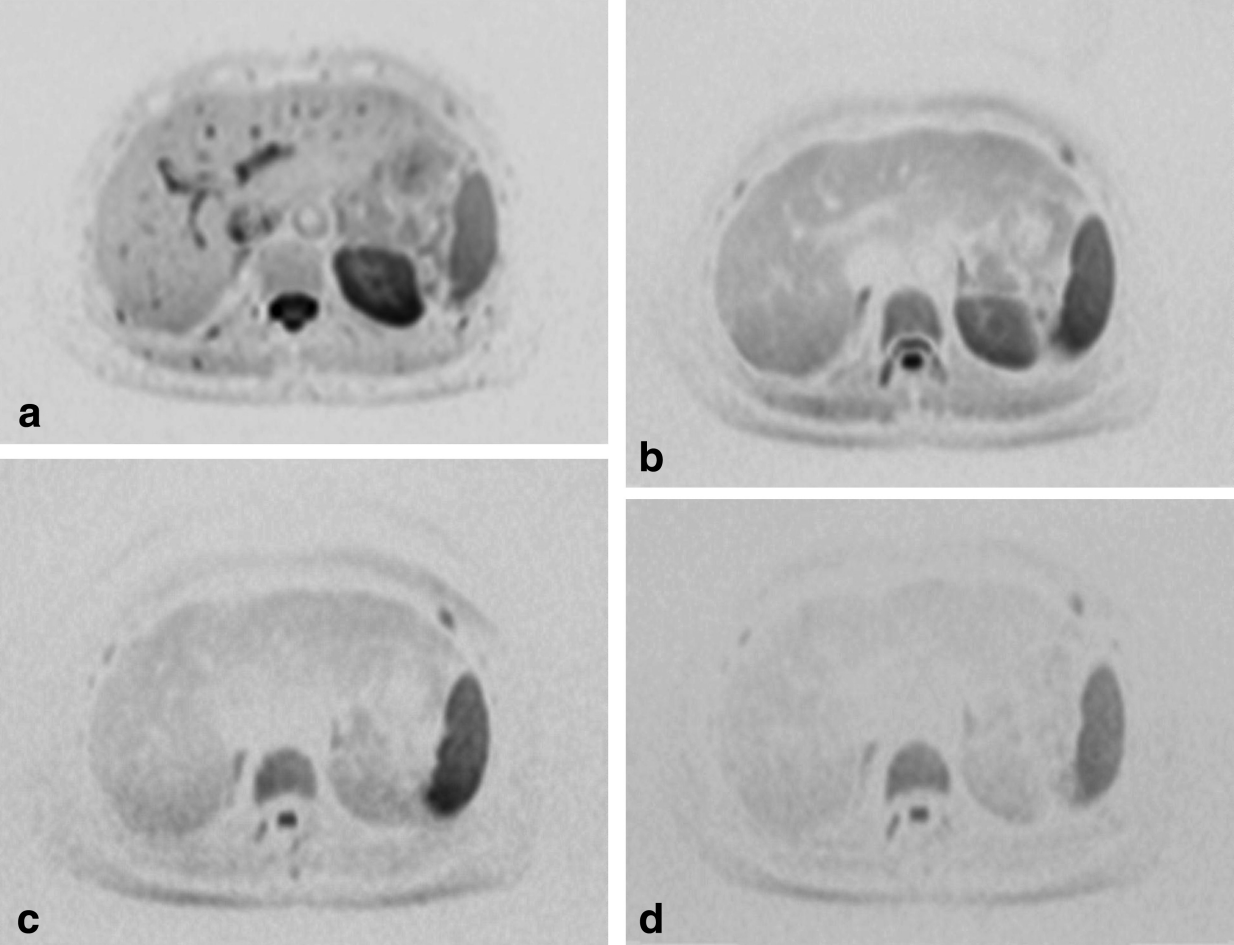
Diffusion imaging of a cross section through the abdomen at b-values of 0 (a), 200 (b), 800 (c), and 1000(d) s/mm^2^. At a b-value of 0, note the dark areas representing fluid, flowing or static. At a b-value of 200, flowing fluids are no longer visible. As b-values increase, there is a resultant loss of background tissue, with minimal normal background liver seen at b-value of 1000 (d).

**Figure 4:**
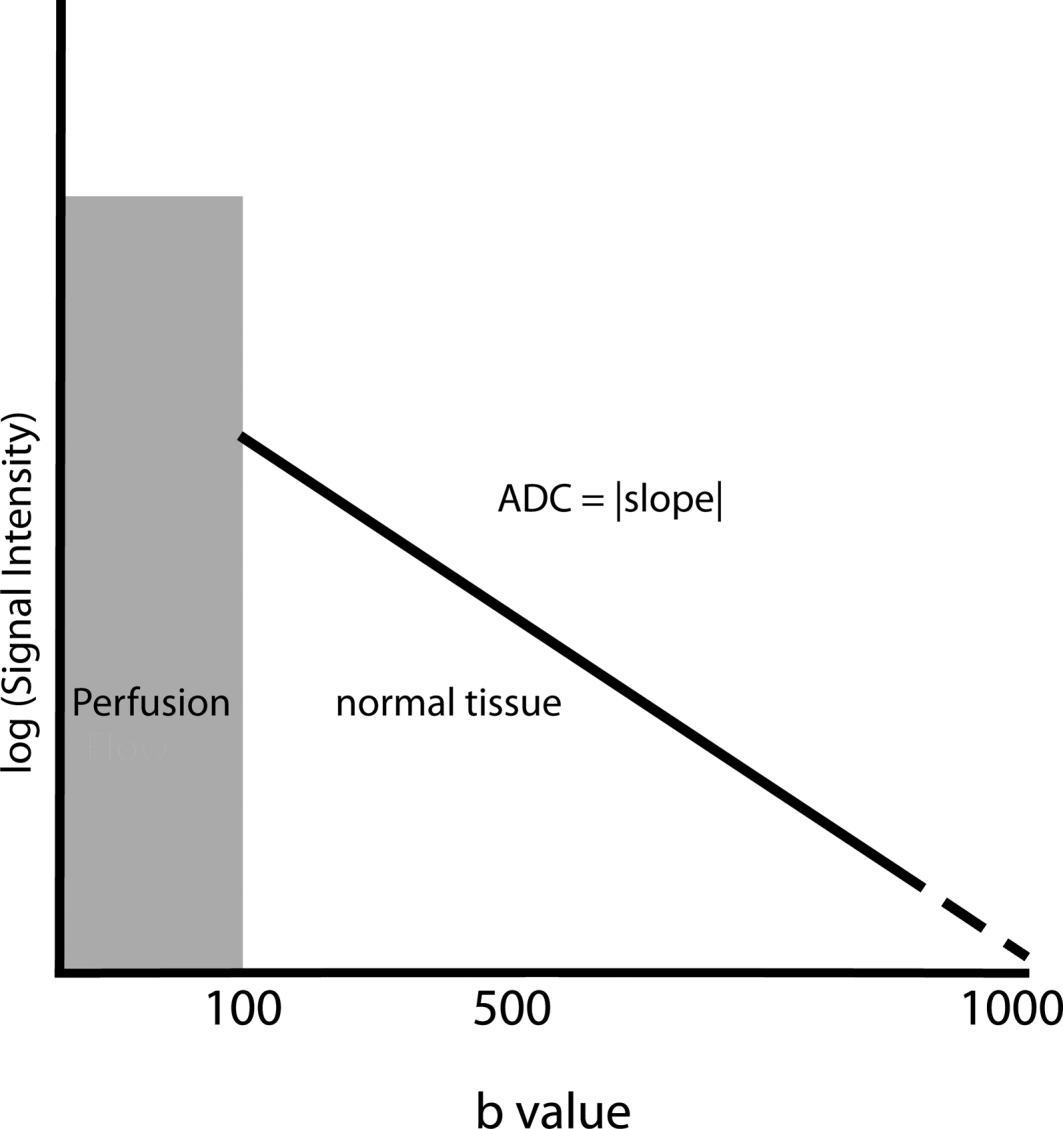
Generic body tissue diffusion curve. Log signal intensity versus b-values for most body tissues show a biexponential behavior with an inflection around b-value of 100 s/mm^2^. At b-values below 100 (shaded), there is a tissue-dependent and additive perfusion flow effect resulting in increased signal intensity which can skew ADC calculations. At b-values above 100, the perfusion effects in the body are limited, resulting in an essentially linear true diffusion slope.

**Figure 5:**
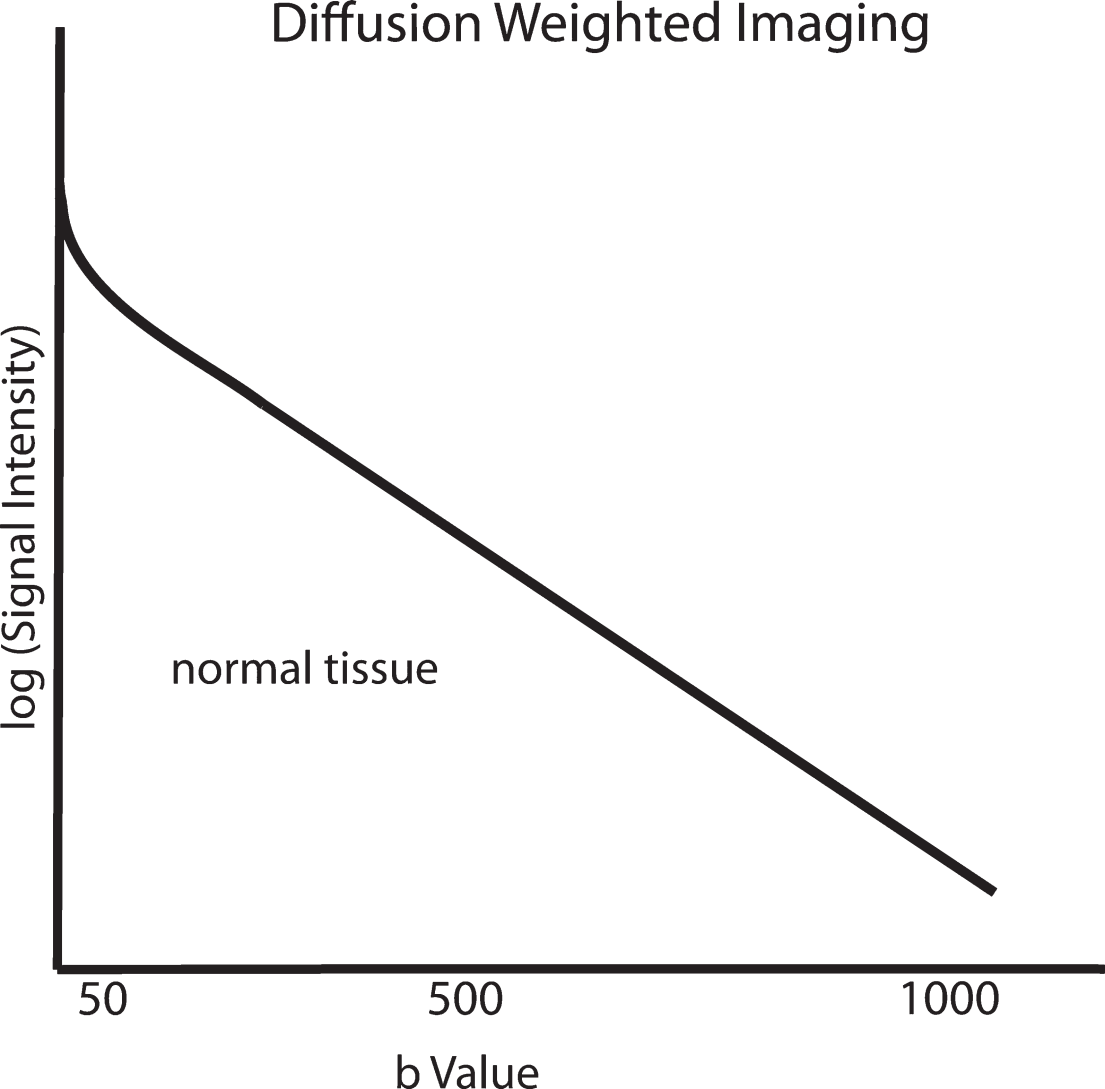
Tissue diffusion IVIM curve. The apparent diffusion (ADC) is the absolute value of the slope of the curve. As higher b-values are used, the background signal decreases.

The log signal intensity versus b-values for a specific tissue can be obtained, and is frequently termed the tissue diffusion curve, which essentially serves as a water-based fingerprint of the tissue being examined 14. The simplest diffusion model assumes tissue is homogeneous, and results in a straight monoexponential line of signal intensity versus b-value. However, the body tissue microenvironment at which water diffusion is measured is inhomogeneous (non-Gaussian), due to cellular compartments, membranes, and vascularity, which result in the tissue diffusion curve being nonlinear. The most pronounced nonlinear effects are due to vascular perfusion, predominantly capillary flow 15,16, and occurs in addition to the bulk diffusion (Figs. 3, and 4]. Thus a bi-exponential tissue diffusion model obtained by a minimum of three b-values resulting in two logarithmic linear components: one for perfusion/flow effects at b-values from 0 to 100 s/mm^2^ and another for tissue b-values above 100 s/mm^2^ for bulk diffusion, can suffice for most clinical applications of diffusion body imaging. Because of the additive effects of perfusion and bulk diffusion, there is a steepening of the slope of the diffusion curve that occurs at b-values less than 100 s/mm^2^ (Fig. 5). Extrapolating and then subtracting the b < 100 s/mm^2^ bulk diffusion component from the diffusion component, a technique known as curve stripping, at b = 0 yields the flow or perfusion fraction which compares well with in vivo experimental data 17. Using multiple b-values can yield further refinements to the tissue diffusion curve, and this is typically referred to as intravoxel incoherent motion (IVIM). When the IVIM curve for a tissue is obtained, the bi-exponential data can also readily be extracted. More complex methods such as kurtosis, which assesses the probability extent of non-Gaussian behavior, and diffusion tensor imaging (DTI) have also been applied to body tissue diffusion investigation.

Obtaining multiple high b-values for IVIM curves is time-consuming and error prone because as b-values rise, signal intensity decreases resulting in loss of lesion to background tissue conspicuity. At high b-values, the relative signal intensity error increases because the signal approaches the noise floor level with resultant poor signal to noise ratio. The lack of standardization, phantoms, or calibration factors results in each machine configuration having its own intrinsic signal to noise, and specific noise floor characteristics.

To aid in lesion characterization, functional imaging techniques are commonly applied; wherein the lesion signal behavior is assessed relative to known tissue. Commonly in the body, this has led to assessing the lesion signal to the tightly regulated anisotropic spinal cord signal 18; yielding the lesion to spine ratio. The signal from the spinal cord effectively acts as an intrinsic calibrating factor, which can be used as a qualitative surrogate for assessment of b-value signal to noise.

The apparent diffusion coefficient [ADC] represents the absolute value of the slope of any two points on the diffusion curve and is calculated (Eq. [2]) using the b-values and signal intensity (SI) (Fig. 5).
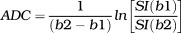
[2]

Most vendors by default use a voxel-based best fit linear monoexponential curve to calculate the ADC, which can lead to considerable error when b-values below 100 s/mm^2^ are used in the calculation, as the effect of perfusion will result in steepening of the best fit curve, and thus an overestimation of the ADC. However, not all vendors allow selection of specific b-values, for ADC and perfusion/flow fraction calculations.

**Figure 6:**
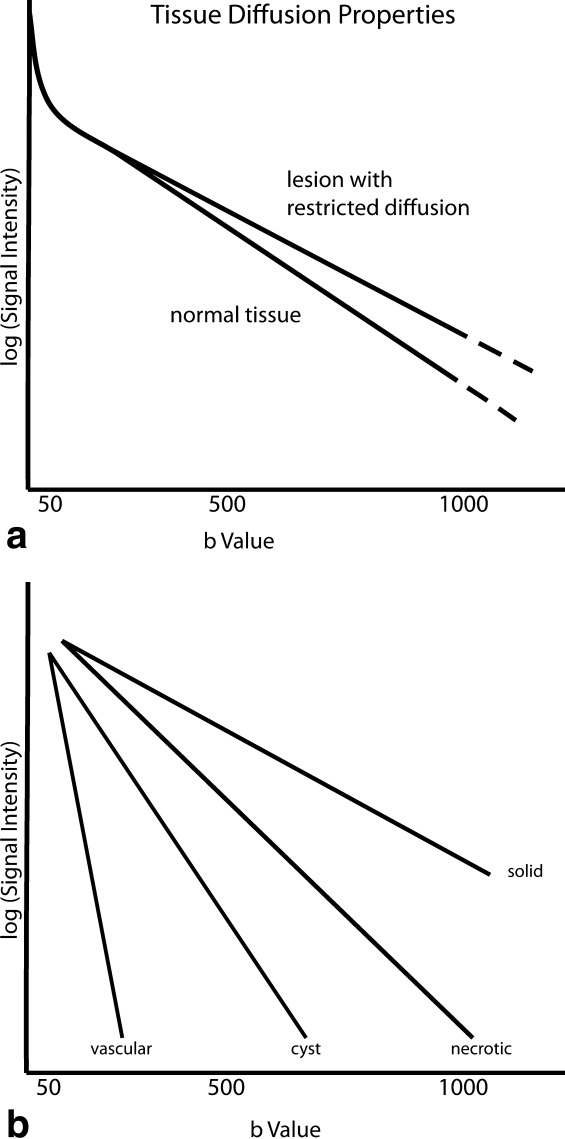
Tissue diffusion curve behavior for native tissue, and a restricted lesion within the tissue (a). The restricted lesion has a less steep slope and thus low ADC value. In body imaging typically the greatest separation in signal intensity between native and restricted tissue with background tissue visibility occurs near b-value of 500 s/mm^2^ (b). Outlines the difference in slope/ADC from near horizontal for solid tissue to the steepest for vascular lesions. The slope differences represent the premise for assessing ADC change of a solid restricted mass initially becoming edematous with treatment with resultant rising ADC values.

Based on the Stokes-Einstein Eq. [1], the impact of changes in tissue water motion, and solvent viscosity can be predicted, yielding increased signal for solid tissue, or viscous fluid. A generalization of expected slopes and signal intensity for tissue encountered in body imaging is shown in Figure 6. Due to lack of standardization, the difference between signal intensity of a restricted tissue versus normal tissue at a fixed b-value cannot be adequately quantified; however, the relative difference can be used to increase lesion conspicuity. This fact can aid with discriminating empyema versus simple pleural effusion 19,20 and peritoneal carcinomatosis versus ascites 21,22, which are difficult to distinguish by conventional sequences. The persistence of increased signal with restricted solid tissue corresponds to the lower ADC values described for solid tumor masses. However, the lack of standardization 23 of b-value selection for various organs and lesions limits the reproducibility of ADC values. A generalization of change in ADC values between vascular, cystic, necrotic/viscous, and solid tissue is outlined in Figure 6b. Thus, the low ADC of a solid lesion undergoing therapy can change. Necrotic or edematous tissue will result in an increase in the ADC value. This quantifiable change, if performed at both baseline and following treatment with the same b-values, can be used to monitor response to treatment.

ADC maps are an image-based mathematical displayed calculation that is subject to inherent errors present in its underlying individual b-value image map components. These errors include inhomogeneous fat suppression, high b-value noise floor effects, movement that can lead to misregistration, edge of field effect, or other gradient/echo planar induced artifacts. Thus to minimize these errors, it is prudent to view the raw b-value images directly in conjunction with conventional coregistered sequences. To detect such errors, it is beneficial to obtain sufficient background signal from the organ in question to be able to localize a lesion. Background signal is rapidly lost at high b-values (greater than 800 s/mm^2^), with large patients, and with inhomogeneous RF maps. Increasing the number of averages to increase signal increases scan time. Near the diaphragm this can magnify errors. Therefore optimal b-value selection should be sought to provide the optimal target to background signal based on machine specific intrinsic resolution.

**Figure 7:**
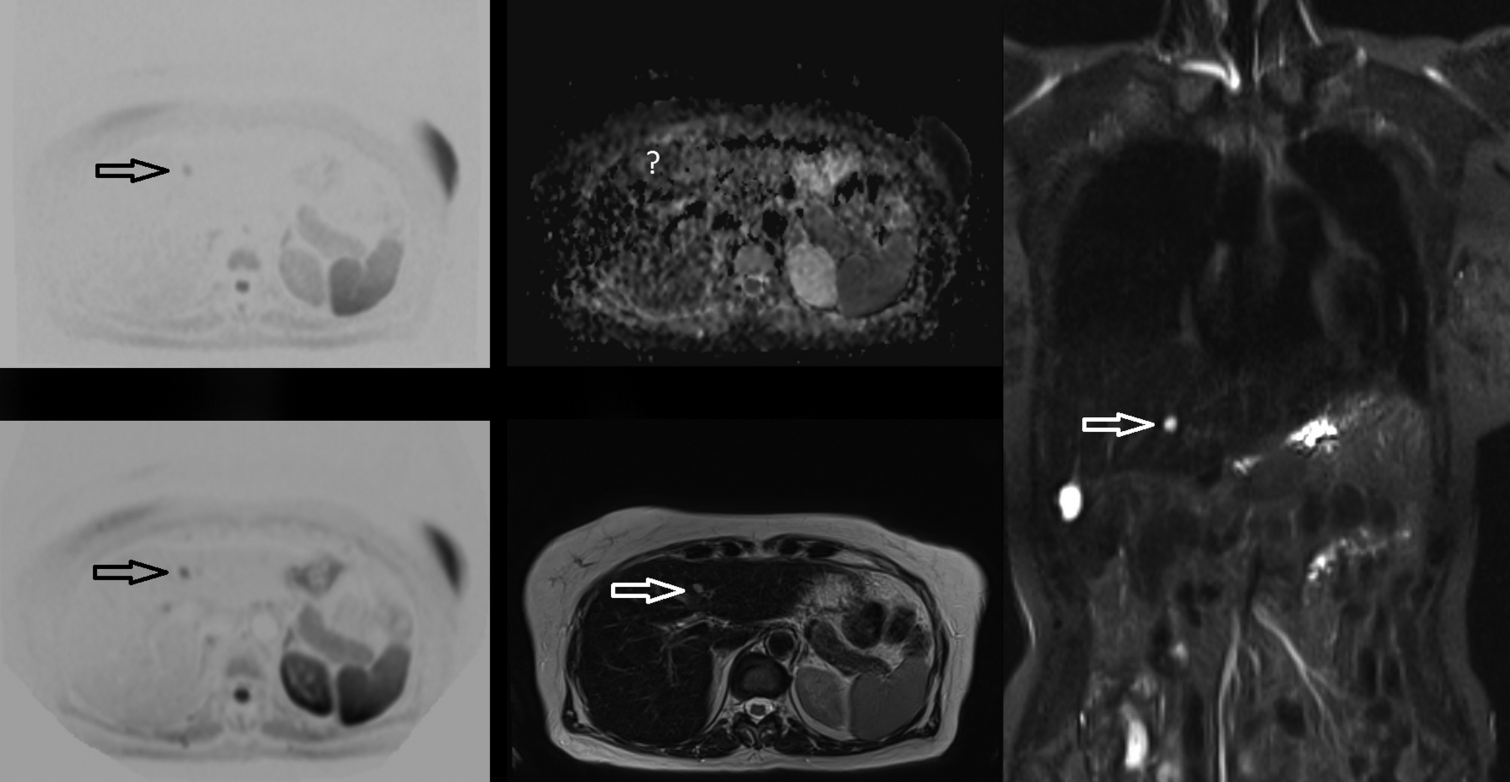
ADC misregistration due to breathing. Upper left b-500, lower left b-50 demonstrates a lesion which is conspicuous from background tissue, with the lesion signal intensity dropping on the b-500 image. The relative signal change is typical for a simple cyst, as confirmed with hyperintense features on the axial T2 (lower middle) and coronal STIR sequences (right). The ADC map (upper center) shows no apparent hyperintense signal characteristic of a cyst due to the misregistration. Note the noise of the ADC map in the liver from breathing movement, which is not as apparent in the left kidney due to less movement.

Misregistration is a common artifact that can occur most commonly at sites of maximum movement (i.e., near diaphragm, where with free-breathing techniques, the b-value maps are not obtained with the organ in question in the exact same position (Fig. 7). This results in ADC map noise, with the risk of loss of detection, or incorrect characterization of a lesion. This potential error can be minimized with diaphragm gating techniques, and stresses the importance of coregistered anatomic routine sequences acquired in conjunction with DWI.

At 3T, the benefit of increased SNR for DWI is offset by an increase in image distortion and artifacts. Field inhomogeneities are compounded, and dielectric effects, incomplete fat saturation and increased eddy currents result in increased DWI signal distortion. These effects can be mitigated by multitransmit RF shimming coils. Rosenkrantz et al 24 have demonstrated no significant difference in ADC values at 1.5T and 3T in matched patients with the same b-values and ADC calculation methods, however, the authors report significantly lower subjective image quality at 3T.

Use of conventional sequences with whole-body imaging at 3T is problematic due to specific absorption rate (SAR) heating effects which are proportional to the square of the static field and the flip angle. Thus the SAR limit is frequently attained at 3T 25. However, the higher SAR requires cooling pauses during scanning 26 which can result in patient motion, and loss of coregistration of sequences with DWI. With multitransmit body coil modeling at 3T, studies have reported mixed results, with modeling reporting both reduced 27 and increased SAR deposition which is increased up to a factor of 13.4 times for focal deposition and 1.6 times for average whole-body scans 28.

**Figure 8:**
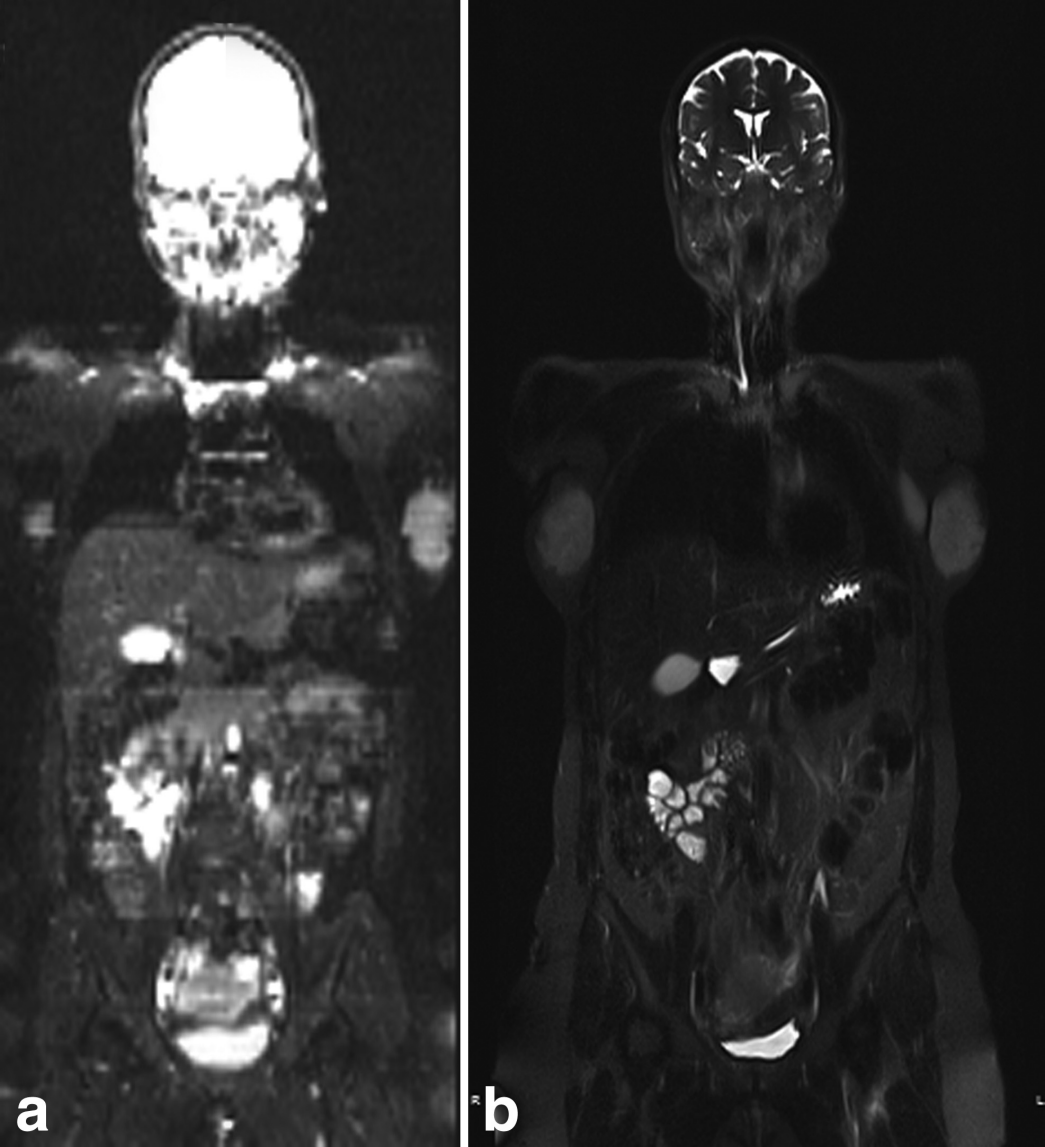
Coronal whole-body images. The b-value image at b-0, shown as white on black, represents a fat saturated fluid sensitive sequence (a), however, the purpose of DWI as a functional technique is to maximize lesion conspicuity by maximizing signal to noise. Typically this is performed with 3 × 3 mm by 5 mm thick slices, as compared with the 1.6 mm × 1.6 mm inplane STIR sequence which demonstrates exquisite anatomic detail (b).

With whole-body DWI (DWIBS) the goal is to maximize lesion conspicuity. Unlike conventional morphologic sequences, where high inplane resolution is sought, with DWIBS, larger voxel size results in the ability to maximize signal to noise. Typically DWIBS is performed at 5-mm-thick slices. The three-dimensional (3D) data set can be reformatted into coronal, and sagittal planes which can be coregistered with conventional morphologic T1- and T2-based sequences. An example of a b = 0 coronal image 3 mm × 3 mm × 5 mm thick voxel is compared with a STIR image obtained with an inplane resolution of 1.6 × 1.6 × 5 mm thick voxel in Figure 8.

Whole-body DWIBS is routinely viewed in inverted grayscale (i.e., black on white) because this format has been found to improve the observer’s inherent retinal contrast resolution in experimental models 29. Similar to other functional nuclear medicine imaging techniques, the black on white versus white on black viewing setting is commonly based on physician preference.

**Figure 9:**
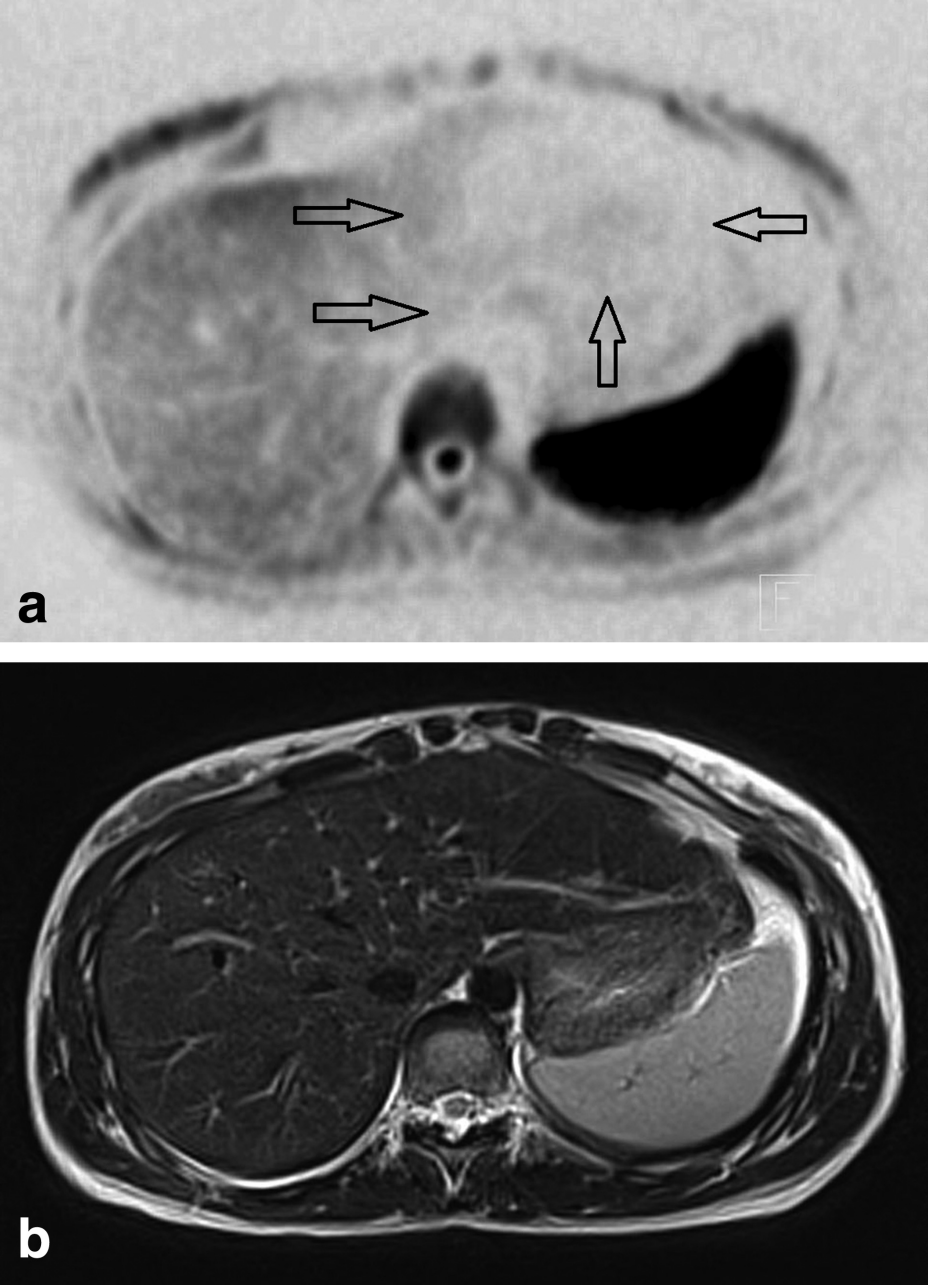
Axial b-500 image (a) demonstrates loss of signal within the left aspect of the liver from cardiac pulsation. The anatomy is not obscured on the axial T2 (b) image. This loss of signal is less problematic at low b-values and can be practically minimized by increasing the number of averages for DWI, or cardiac gating.

Like all free-breathing techniques, whole-body DWIBS suffers near the diaphragm, particularly in the left lobe of the liver where cardiac pulsation results in signal loss as shown in Figure 9. This loss of signal can be minimized by cardiac and/or respiratory gating, and lower b-values. However, for whole-body DWIBS, quiet breathing is preferred to gating techniques to minimize scan time thus maximizing patient comfort. This free-breathing technique has been shown by Takahara et al to result in minimal signal loss 12.

**Figure 10:**
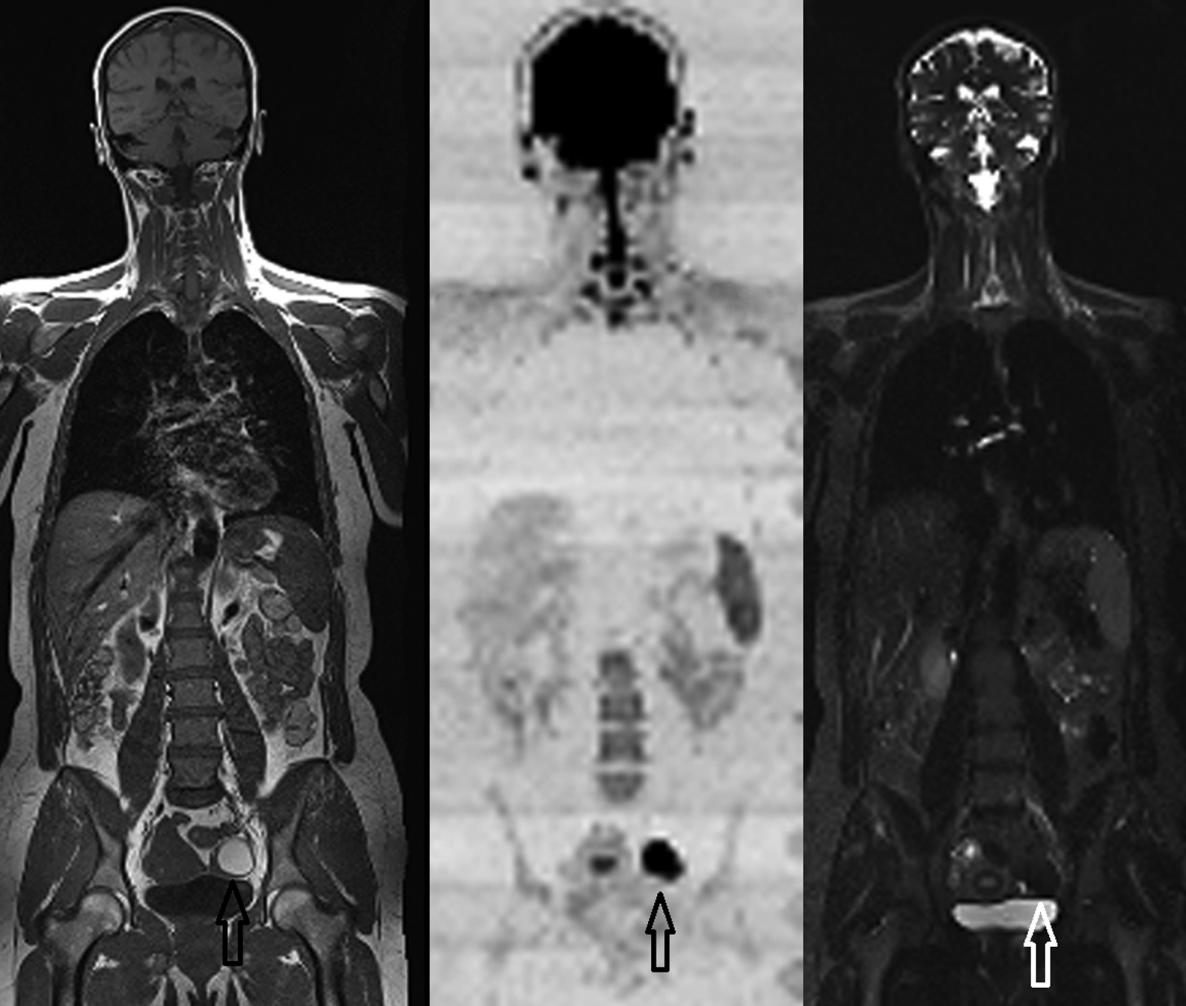
Whole-body coronal T1, DWI (b-500 shown as black on white), and STIR images demonstrating the conspicuity of the left adnexal subacute hemorrhage in an endometrioma due to the paramagnetic effects and restricted diffusion of blood degradation products.

The capability of combining conventional anatomic sequences with a functional technique (DWIBS) in one imaging tool shows promise to enable MRI with DWIBS to parallel the diagnostic advancements demonstrated by positron emission tomography/computed tomography (PET/CT). Combining MRI’s unique power of characterizing tissue based on water and fat content (i.e., T1 and T2 weighting) with DWI’s ability to assess tissue water motion and ADC provides a unique set of noninvasive imaging tools to both detect, and characterize lesions within tissue.

Clinical diffusion-weighted images are T2-weighted; thus, the native tissue being interrogated can elicit T2 shine through effects. Thus, to separate restricted diffusion tissue from T2 shine through effects, methods analogous to those applied to the brain can be used, namely comparison with standard T2 sequences, and with ADC maps/values. Normal tissue, which demonstrates restricted diffusion due to anisotropy, or tissue density include brain and spinal cord, spleen, lymph nodes, red marrow, ovaries, testes, and endometrium. Restricted tissue based on T2 shine through include normal gallbladder, salivary glands, prostate, and hemangiomas 30. Early hematomas in the body demonstrate similar findings to those shown in intracranial hemorrhages and appear restricted on DWI imaging because of both paramagnetic effects and restricted diffusion 31; thus, the importance of comparison with conventional sequences for appropriate tissue assessment is demonstrated in an example of an endometrioma (Fig. 10).

Bone marrow changes with age, disease, and post-chemoradiation is well demonstrated with PET/CT and skeletal scintigraphy, with DWIBS demonstrating analogous findings. Youth and marrow expansion conditions have increased red marrow within the tubular long bones, that shrinks with age to involve predominantly the axial skeleton. Hematogenous red marrow is known to have high cellularity giving restricted signal on DWI compared with yellow/fatty marrow, which has up to 70% less water 32. Thus, assessing marrow disease or infiltration from metastatic disease or multiple myeloma can be readily detected by DWIBS. Stecco et al 33 found DWIBS to have the same specificity as skeletal scintigraphy, with bone scintigraphy demonstrating limitations in the pelvis, coccyx, and sternum. DWIBS was found to show a higher specificity, but lower sensitivity with NaF PET/CT in patients with prostate bone metastasis 34. This lower sensitivity is likely due to the paucity of water within cortical bone; thus, cortical lesions may result in false negative findings on DWI.

DWI performed postgadolinium administration has been shown to have a similar appearance to DWI performed pregadolinium in the prostate, liver, spleen, pancreas, and kidney 35–37.

Whole-body imaging is commonly used as a survey for multisystem symptoms, and to determine metastatic spread or distant disease burden. This ability to perform a whole-body survey has been an advantage of scintigraphic nuclear medicine techniques, with colocalization from CT adding the benefit of lesion conspicuity to soft tissue background. DWIBS coregistered to morphologic MRI techniques provides similar lesion detection and localization capabilities without the radiation used by other techniques. MRI also has the advantage of improved soft tissue characterization. Through the combined interpretation of morphologic routine images and functional DWI, the sensitivity and specificity of whole-body MRI is maximized 38–40. Whole-body MRI with DWIBS is considered as an alternative tool to conventional whole-body methods for tumor staging and during follow-up in colorectal, myeloma, Hodgkins lymphoma, lung, and breast cancer patients with the difference between DWIBS and gadolinium contrast enhanced T1 MRI showing no statistical significance, with both techniques being comparable to FDG PET/CT 41. A meta-analysis of 1239 patients with whole-body MRI and FDG PET/CT comparing metastatic disease sensitivity found similar lesion detection capabilities between the two modalities 42.

To harness the maximum benefit of DWIBS and morphologic routine images, correlation with fat and water based high in plane resolution whole-body sequences such as T1 and STIR can be used. Five-millimeter-thick sections with DWIBS, T1, and STIR of the whole body can be acquired in approximately 40 min at 1.5T, with machine specific DWIBS protocols provided elsewhere 13.

Whole-body MRI conventional techniques coupled with DWIBS results in detection capabilities and characteristics for many oncologic conditions that are similar to that of FDG PET and bone scintigraphy, while adding the capability of allowing quantitative tumor volumes and response to therapy in new areas such as skeletal metastases, multiple myeloma, and lymphoma. These whole-body techniques by their inherent imaging volume allow improved repeatability and precision 43–46, which aid in assessment of response to therapy. The time and technique necessary to review a whole-body DWIBS with coregistered T1 and STIR is similar to that for a comparable PET/CT study. The overall approach to lesion quantification for diagnoses and follow-up is also analogous between standard uptake values (SUVs) of PET, and those of ADC measurements.

The vast majority of body DWI investigations have been performed at an individual organ specific level, which commonly include multiple varied, and often institution specific techniques. Thus, whole-body MRI techniques should typically not be used to supplant organ specific detailed MRI studies. To appropriately interpret DWIBS, as with all whole-body imaging modalities, a rigorous understanding of organ-based signal changes with disease processes is required.

## DWI BODY APPLICATIONS

### Neck

DWI applications to the head and neck are promising 47; however, the lower neck and shoulder region suffers from RF field inhomogeneity, which can induce frequent artifacts and edge of field errors. These can be overcome by imaging the region of interest in isocenter, and limiting the z axis field of view. Low b-values suffer from perfusion effects which may also include secretion effects from glandular tissues.

Thyroid nodule assessment for discrimination of benign versus malignant conditions has been performed in multiple studies. Mutlu et al 48 found using b-values of 0, 50, 400, and 1000, with comparison to the spinal cord at b-value of 1000 in 44 patients were able to achieve a specificity of 97% and accuracy of 98% compared with fine needle aspiration, with Nakahira et al 49 using an ADC cutoff of 1.6 × 10^−3^ s/mm^2^, demonstrating a specificity and accuracy of 83% and 88%, respectively.

DWI has been shown to improve primary lesion detection in squamous cell carcinoma and can aid in histologic grading and nodal spread compared with conventional CT, MRI, and PET 50–54, with preliminary studies incorporating DWI demonstrating favorable prognostic results for disease-free survival 55,56.

### Lung

Discrimination of atelectasis from an associated pulmonary mass can be attained by visual inspection of the multiple b-value images, with the tumor mass demonstrating lower ADC than the adjacent collapsed lung 57.

A study by Regier et al 58 at 1.5T demonstrated that respiratory gated DWI compared with the gold standard of multidetector CT was 97% sensitive for detection of pulmonary nodules greater than 1 cm in diameter, and 86% sensitive for nodules measuring 6–9 mm in diameter. MRI and FDG PET are both susceptible to image blur, and resultant loss of resolution with free-breathing acquisition near the diaphragm when compared with the lung apex, where there is less lung movement with respiration.

In 2009, Ohba et al 59 performed a prospective study on solitary pulmonary nodules using both DWI and FDG PET, using surgical specimen histology as the reference standard. This study demonstrated similar sensitivity, with DWI being more specific than PET for nonsmall cell lung carcinoma, with Regier et al 60, demonstrating an inverse correlation with standard uptake values (SUV) and ADC values calculated from b-values of 0 and 500. Nodal assessment using DWI shows either similar results, or modest improvement compared with FDG PET 61–63.

### Breast

Breast tissue demonstrates substantial heterogeneity, with women of childbearing age having predominantly glandular tissue, which limits mammographic sensitivity. Postmenopausal women have unique breast density ranging from almost entirely fat through which mammograms are well suited, to extremely dense breasts. DWI has been applied as a possible technique to aid in diagnosis of breast cancer detection. Many studies have been performed using combined dynamic contrast enhanced MRI in conjunction with DWI. These tests find variability in DWI sensitivity for detection of breast carcinoma from normal fibroglandular tissue, with ADC variations occurring across the menstrual cycle 64. Using b-values of 0 and 600, Partridge et al 65 have described a sensitivity of 96% and specificity of 55% for discrimination of benign versus malignant breast masses. The sensitivity and specificity is improved adding dynamic contrast enhancement, secondary to a vascular component within breast lesions 66,67. Intravoxel incoherent motion analysis on women with breast cancer has been performed by Sigmund et al 68, and these studies have described a biexponential shape of the diffusion curve in breast lesions. In women under 50, who most commonly have heterogeneously dense breast tissue, Kazama et al 69 demonstrate DWI using b-values of 0 and 800, combined with mammography results in significant improvement in malignancy sensitivity of 93% versus mammography alone of 64% with insignificant specificity loss (85% versus 92%). Future studies examining perfusion, and diffusion ADC values in addition to mammography would be beneficial to ascertain the possibility for developing improved noninvasive lesion detection.

### Genitourinary

**Figure 11:**
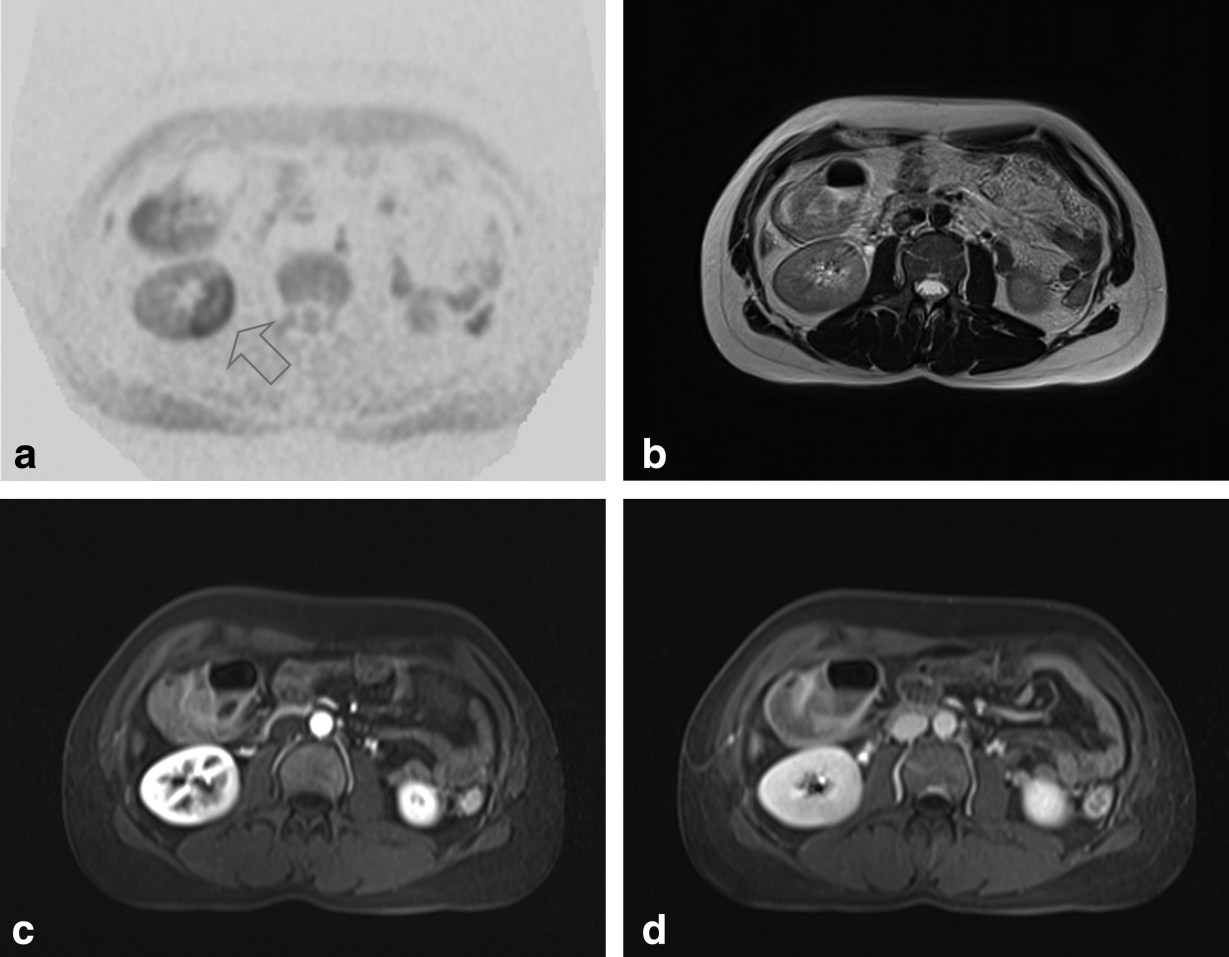
Axial b-500 (a) readily demonstrates the increased signal from localized pyelonephritis, which cannot be identified on axial T2 (b) images. The subtle loss of arterial phase enhancement from pyelonephritis is shown in (c), with near complete loss of lesion conspicuity on delayed contrast enhanced images (d).

DWI provides valuable functional information about lesions throughout the kidney, which can be seen to greater advantage than by conventional anatomic techniques. The cortical renal changes that occur in early childhood demonstrate increasing ADC with age, with the most rapid change occurring in the first year of life 70. The effect of pyelonephritis can readily be identified by DWI 71,72, without the use of contrast material (Fig. 11), whereas the findings by anatomic imaging and vascular enhancement can be subtle. The increase in viscosity of pyonephrosis can be demonstrated by a decrease in ADC values within the renal collecting system 73.

Chronic renal failure patients have been found to have significantly lower ADC values than patients with normal renal function 74. The kidneys have a bi-exponential curve, with both perfusion and diffusion components. With decreased renal perfusion from renal artery stenosis and acute ureteric obstruction, the cortical perfusion component (b-value < 100) has been shown to decrease 75. Separating acute renal failure from chronic renal failure, and correlation with glomerular filtration rate shows promise, with acute renal failure likely having a small decrease in ADC within the renal cortex, whereas chronic renal failure demonstrates a large decrease in ADC 74. In a small series of 4 patients within 19 days of renal transplant who demonstrated acute rejection, the marked decrease in perfusion measured by DWI correlated with creatinine clearance 76.

Investigation of renal cell carcinoma (RCC) discrimination from simple cysts and complex cysts has been undertaken by numerous studies, with nearly all incorporating b-values of zero into their linear monoexponential calculation of ADC values 77–80. Nonetheless, these studies are able to separate cystic fluid from solid mass by the marked difference in ADC values, with some authors using DWI to attempt to correlate subtype of RCC’s with histologic subtype 81,82. Zhang et al 83 found by retrospective review of the literature, that the lesions in the kidney were best described by an IVIM biexponential model, with further optimization techniques also being possible to maximize precision and accuracy 84. Incorporating both perfusion and diffusion for assessment of enhancing renal masses, Chandarana et al 85 found improved characterization of renal masses, with the tumor mass DWI perfusion components having good correlation with lesion vascularity, which may be beneficial in situations where contrast cannot be administered.

In patients with gross hematuria, DWI with b-values of 0 and 800 in conjunction with T2 images have been used to successfully identify bladder carcinoma with a sensitivity of 98% and a specificity of 92% 86. DWI in conjunction with T2 improves discrimination of superficial lesions from bladder invasion to 64% from 40% using T2 sequences alone 87, with several studies demonstrating ADC values correlating with histologic grading 88–90. With imaging for bladder tumors incorporating DWI, Takeuchi et al 91 were able to detect foci of restricted diffusion within the ureters and/or urethra that corresponded to synchronous tumors, or were from clot, with the signal intensity being secondary to the paramagnetic effect of blood detected by DWI. DWI has also been shown to separate postsurgical bladder wall inflammatory changes from bladder tumors 92.

**Figure 12:**
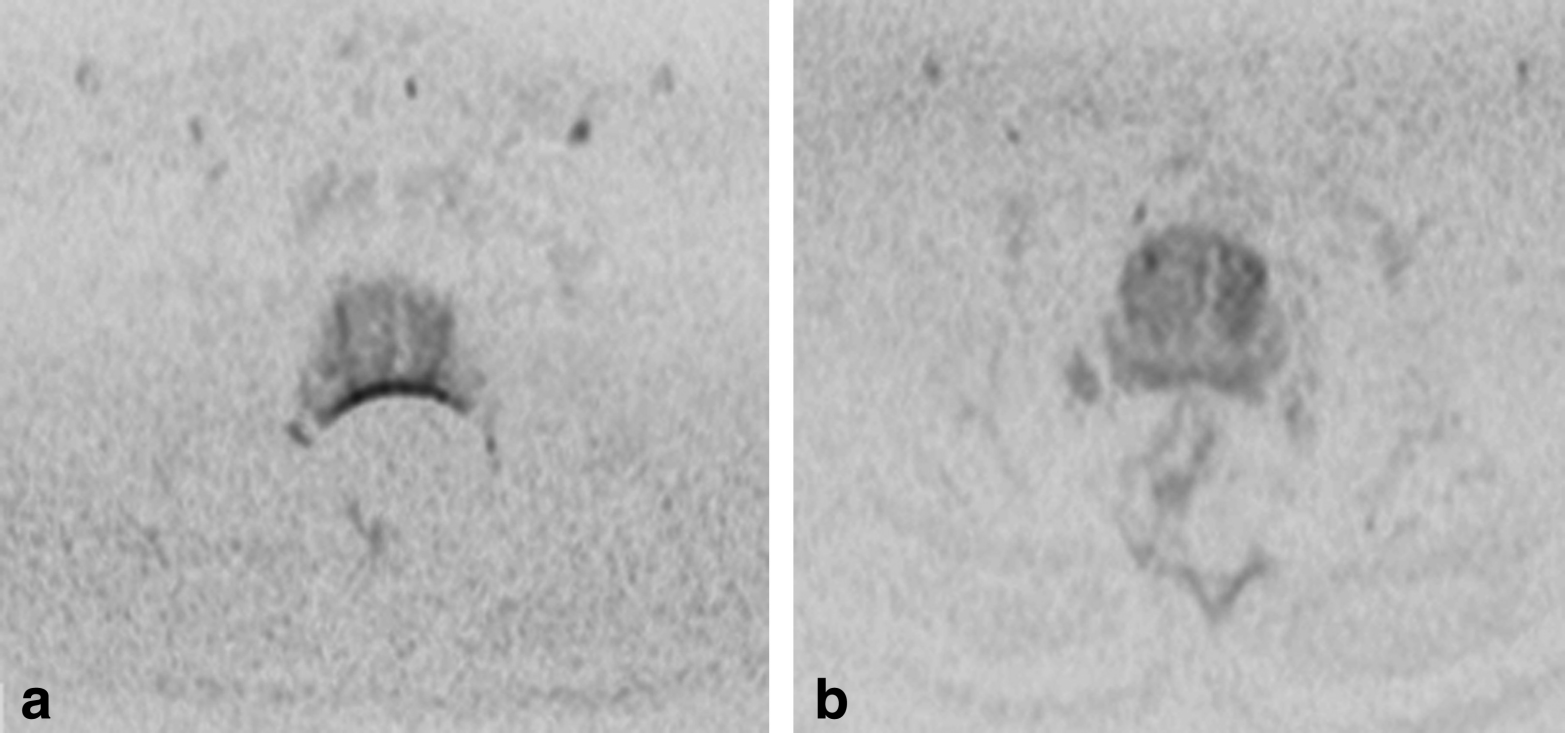
Prostate imaging. DWI b-800 demonstrates marked susceptibility from gas in the rectum (a) obscuring visualization of the peripheral zone. This can be simply rectified noninvasively by prone positioning (b) of the patient, or with rectal gel.

Innumerable studies using DWI have been applied to the prostate gland, largely owing to the invasiveness, and high false negative rate of systematic ultrasound-guided biopsy. The central location of the gland within the body, and variably small size of the peripheral and transitional zones with age makes MRI assessment challenging. Applying a functional imaging technique in conjunction with routine morphological sequences has yielded improved tumor localization; however, the variability in imaging parameters, coil selection, and field strength without standardization makes comparison of results difficult. Due to the complexity of these problems, multiparametric assessment of the prostate gland has shown the most promise. These techniques include conventional anatomic sequences (T1 and T2), in addition to functional techniques of DWI, dynamic contrast enhanced MRI (dceMRI), which assesses perfusion, and H^1^ spectroscopy. A meta-analysis of 5892 prostate lesions has shown that DWI and T2 combined have a higher sensitivity and specificity (0.73,0.83) than dceMRI (0.58,0.82) alone 93. High b-values used in ADC calculations have shown the greatest promise in lesion localization 94,95; however, higher b-values suffer from loss of signal with an increase in noise (Fig. 4). DWI applied to the prostate can suffer greatly from inhomogeneous RF map which is magnified at higher b-values, and can limit evaluation of the prostate gland. This can be pronounced from gas in the rectum (Fig. 12), or from a poorly coupled endorectal coil. The latter of which also distorts the morphology of the prostate. Hoeks et al 96 for detection and tumor localization, suggest the minimal requirements for multiparametric imaging of the prostate is a combination of T1 and T2 sequences with either DWI or dceMRI using only a pelvic phased array coil, however, the addition of an endorectal coil is suggested for staging.

Assessment of perfusion in the prostate is commonly performed using intravenous gadolinium dceMRI; however, IVIM comparison studies 97 have shown promise in separating the perfusion from the diffusion components at low b-values 98. Higher order diffusion modeling (kurtosis) has shown further improvement in defining the non-Gaussian characteristics of the prostate gland 99, and ADC values have been shown to aid in tumor aggressiveness 100,101. Further improvement in tumor ADC utility within the peripheral gland has been shown in a small sample by accounting for the interpatient variability of ADC values in normal peripheral zone with a resultant nomogram developed to aid in risk assessment of tumor aggressiveness 102.

### Gynecologic Applications

DWI has been found to be useful in detection of diffuse infiltrating endometriosis, by using the restricted diffusion from fibrotic tissue deposition 103 and paramagnetic effect from degrading hemoglobin to aid in lesion detection (Fig. 7). DWI with ADC values has been shown to separate endometrial cancer from normal endometrium and myometrium using 1.5T with a body array coil and b-values of 0 and 800 104. DWI has been shown to separate cervical carcinoma from normal cervix at 1.5T with a body array coil and only two b-values of 0 and 800 105. Using multiple b-value combinations (0, 150, 500, and 1000), Hoogendam 106 at 3T with a torso coil demonstrated all combinations of b-values are able to separate IB and IIA cervical carcinoma from normal cervical tissue. Tissue typing and histologic grading correlation with IVIM and multiple perfusion b-values has yet to be evaluated for cervical carcinoma; however, parametrial invasion and tumor extension is typically well characterized by conventional sequences.

Ovarian carcinoma is typically diagnosed noninvasively by conventional anatomic MRI techniques, and owing to the heterogeneity of ovarian mass tissue, the contribution of DWI is limited. However, recent studies have demonstrated incorporating dceMRI and DWI (b-value 0, 500, and 1000) yielded an improved accuracy of 95% in characterizing complex adnexal masses as benign or malignant. These functional techniques resulted in change in initial diagnosis ranging from 19 to 24% per reader compared with conventional sequences alone 107. Multiple b-values to evaluate the perfusion component in ovarian cancer has been assessed and demonstrates the capability to replace contrast enhancement in patients who are unable to have gadolinium 108,109. Additionally multiple b-value assessment has shown the difference in the perfusion component between ovarian primary, omental cake, and peritoneal carcinomatosis. Only changes in ADC of the ovarian mass occur with treatment. Other nonovarian malignant tissue diffusion characteristics remain unchanged 110. The stability of the peritoneal carcinomatosis and omental DWI findings are likely secondary to viscosity changes, rather than tumor vascularity changes being detected by DWI.

### Esophagus, Stomach, and Bowel

A paucity of data is available in the literature regarding using DWI for assessment of the esophagus, stomach, and bowel. This may be due to the difficulty with susceptibility and motion from the gut. A single study reveals the detection capability of gastrointestinal stromal tumors (GIST) with DWI using oral water with the ability of the ADC (b-values of 0 and 1000) being able to differentiate good responders from treatment poor responders. In this study, initial pretreatment ADC values for poor responders was 1.24 versus 1.06 [× 10^−3^] s/mm^2^ for good responders, with the good responders demonstrating response to therapy by 1 week, characterized by an increase in ADC value to a mean of 1.60 [× 10^−3^] s/mm^2^ 111. DWI alone has been shown to aid in stomach mass lesion detection, with the possibility to discriminate between adenocarcinoma and lymphoma 112.

In small studies, DWI has been used in assessment of the esophagus with limited success, likely due to cardiac pulsation, and respiratory motion masking signal of the lower esophagus. In a trial of 24 patients, Sakurada et al 113 found a 49% primary tumor detection rate, with nodal group sensitivity of 39% and specificity of 93%. Aoyagi et al 114 found a correlation with restricted ADC values and stromal collagen in a small prospective esophagectomy trial of 17 patients.

Patients with inflammatory bowel disease are frequently assessed with imaging and colonoscopy. Most imaging is composed of CT scans; however, due to the young age of the population, there is rising concern about the long-term risks of CT radiation. Additionally, the invasiveness of colonoscopy, and the requirements for bowel preparation are onerous for patients. In a study comparing DWI (b-value 0, 600), gadolinium enhancement, bowel thickening, and colonoscopy in 35 patients with ulcerative colitis, and 61 with Crohn’s disease, Oussalah et al 115 found DWI to be as sensitive and specific for detecting segments of active inflammation as contrast enhanced MR enterography, without the need for oral or rectal preparation/contrast. Additionally these authors found DWI alone to be able to reliably detect inflammation in patients with ulcerative colitis. DWI (b-value 0, 50, 800) was found to be helpful in small bowel Crohn’s detection with a free-breathing technique, with ADC values being lower in affected segments 116. In a small study (n = 23) Kilickesmez et al 117 found DWI (b-value 0, 500, 1000) ADC values trending progressively lower from normal rectosigmoid colon, ulcerative colitis to lowest in adenocarcinoma, with overlap. ADC values in patients with ulcerative colitis of the rectosigmoid were also found to rise and normalize in patients responding to treatment 118.

Using b-values of 0 and 1000, Ichikawa et al 119 were able to assess colon primary carcinomas ranging in size from 2 to 7 cm, with a sensitivity of 91% and specificity of 100%, compared with postsurgical excision. Other studies using b-values of 50 and 800 have been used to assess rectal carcinoma ranging in size from 1.1 cm to 2.5 cm 120. The bowel, particularly the rectosigmoid colon, appears to be most sensitive to susceptibility artifact; however, by repositioning, this can be minimized (Fig. 2).

### Pancreas

Evaluation of the pancreas with DWI continues to evolve. Examination of native pancreatic tissue demonstrates variation in ADC with location, age, and degree of fatty infiltration, with the tail having slightly lower ADC than the head and body 121,122. Variability of b-value selection, and thus ADC calculation has variable discrimination of normal tissue versus mass forming pancreatitis and pancreatic adenocarcinoma 123–125. These difficulties are compounded at 3T, where DWI is found to be of limited value 126, with slight improvement using ratios of lesion to background in assessing focal cystic pancreatic lesions 127. However, IVIM analysis at 1.5T yields differences in the perfusion component, with normal pancreas having the highest perfusion, which significantly decreases with mass forming pancreatitis, and further significantly decreases with adenocarcinoma 128. At 1.5T, using the perfusion component alone, pancreatic adenocarcinoma can be discriminated from normal tissue with a reported sensitivity and specificity of 96% and 100%, respectively 129, versus 92% and 97% using free-breathing and ADC of b-values of 0 and 500 alone 130.

Pancreatic endocrine tumors are a rare variety of tumor, representing only 1 to 2% of pancreatic masses. Typically these are hypervascular on arterial phase contrast enhanced MRI; however, a small (n = 18) retrospective study using fusion of T2 with b-value of 1000 on a 1.5T scanner showed a significant improvement in lesion detection compared with b-values alone 131. This finding is likely secondary to the loss of background tissue visible at higher b-values.

### Liver

Many studies have been performed using DWI in the liver. These studies seek out diffusion characteristics to differentiate the liver based on fatty infiltration, cirrhosis, benign versus malignant liver primary tumor masses, hepatic metastases, and response to treatment. The variability of study design, equipment hardware, and b-value selection makes comparison of the individual studies difficult; however, trends are becoming apparent. Holzapfel et al 132 demonstrate that the conspicuity of lesions identified by DWI can be subcentimeter in size using respiratory gating at 1.5T with ADC values aiding in lesion characterization. Kim et al 133 demonstrate similar findings comparing small hepatocellular carcinomas (HCCs) detected on DWI alone versus rapid contrast washout, with other authors demonstrating DWI in conjunction with conventional sequences results in improved diagnostic sensitivity and reader confidence detecting small HCCs in cirrhotic livers 134. Park et al 135 suggest DWI alone is a reasonable alternative to contrast enhancement for lesions greater than 2 cm in size.

In general, the tissue composition (fluid versus semisolid/necrotic versus solid) results in progressive horizontal flattening of the diffusion curve, with the ADC values consequently dropping (Fig. 6b). Simple cysts, owing to their fluid nonvascular (and thus lack of perfusion) state have the highest ADC values, with no overlap between malignant primary liver lesions; however, overlap with necrotic metastases or treated lesions may occur. Hemangiomas have been shown to have ADC values near that of adjacent normal hepatic parenchyma 136,137, and choosing the cutoff 1.6 [× 10^−3^] s/mm^2^ proposed by Parikh et al 138 would separate hemangiomas from other liver lesions. The perfusion component of hepatic lesions was examined by Yamada et al 139 in an IVIM study, which revealed capillary flow in hemangiomas to contribute the highest perfusion followed by normal liver, metastases, then HCCs.

Fibronodular hyperplasia (FNH), and hepatic adenomas (HA) are easily detected by DWI; however, characterization of these mainly benign lesions by ADC value alone is limited secondary to overlap with malignant conditions 140. FNH lesions, while vascular, have yet to be examined by IVIM methods. Agnello et al 141, using b-values of 0, 150, and 600 found ADC values for FNH and HA to be significantly lower than adjacent normal liver, and they found by taking a ratio of lesion ADC to 15% below adjacent liver ADC resulted in appropriately characterizing 90% of FNH and 80% HA as benign.

DWI with IVIM have been used to assess liver fibrosis, and at 1.5T have demonstrated a decrease in ADC values ranging from 12 to 18% with liver fibrosis compared with normal controls 142,143. The IVIM studies demonstrate both a decrease in liver perfusion and true diffusion resulting in magnification of the drop in ADC. These findings correlate with drop in perfusion measured by dceMRI 143.

Malignant hepatic lesions such as HCC and metastases are readily visualized by DWI, with HCC demonstrating the lowest ADC values of all hepatic lesions 137, due to the low perfusion and restricted diffusion. Nasu et al 144 demonstrated no change in the low ADC values with degree of HCC differentiation.

After treatment of malignant disease in the liver, ADC values have been shown to change, typically before morphologic changes in tumor mass are demonstrated. Several authors 137,145 report their observational findings of increasing ADC with embolization and chemotherapy from cell lysis, with decreased ADC from antiangiogenic drugs, possibly due to impeded blood flow and reduction of extracellular volume.

## CONCLUSION

DWI is a valuable functional imaging technique that can readily be applied to MRI of the whole body. Similar to all other combined anatomic and functional imaging techniques, DWI and conventional sequences demonstrate that the sum is greater than the individual parts, with DWI aiding in both lesion detection and characterization. Body tissue signal characteristics at various b-values is more complicated than simply obtaining a linear ADC value, with most tissues in the body having a perfusion component superimposed on top of the bulk tissue diffusion curve. Thus, calculating ADC values without extracting perfusion from the true diffusion component is rife for error, and this has led to great variability in quantifying tissue properties in the reported literature. IVIM studies applied to the body have demonstrated that using a cutoff b-value of 100 s/mm^2^ can reliably separate perfusion from bulk diffusion; with diffusion predominating at b-values greater than 100 s/mm^2^. Using two b-values at or above b-100 s/mm^2^ can then be used to more reliably calculate an ADC value. To minimize the chance of misregistration, and thus incorrect ADC map, it is ideal to choose b-values that maintain some background tissue signal. Typically b-values of 500 s/mm^2^ up to 800 s/mm^2^ demonstrate maximum separation between native healthy tissue and restricted (hypercellular or viscous) tissue, while maintaining background tissue visibility. The background tissue visibility is important for coregistration with other conventional sequences, and is especially important for free-breathing acquisitions. As no standards exist for measuring or calibrating machine specific intrinsic resolution and noise floor, it is important to choose b-values that yield adequate target to background ratios to confidently assess lesions.

Despite the variability of reported b-values for ADC calculation, using DWI in body applications for lesion characterization in conjunction with T1-and T2-based sequences rivals or supersedes most other non-invasive imaging techniques, with low b-value(< 100 mm/s^2^) perfusion fraction demonstrating capabilities similar to perfusion dceMRI. Once standardized b-values are used to obtain both diffusion, and perfusion information from body imaging, DWI may limit the necessity for gadolinium contrast enhancement. DWI techniques coupled with anatomic conventional morphologic techniques will greatly aid in tissue characterization and reader confidence. This technique allows greater lesion conspicuity and characterization compared with other functional and anatomic imaging modalities, all with the benefit of no radiation.
